# Medications used in pediatric cystic fibrosis population

**DOI:** 10.31744/einstein_journal/2018AO4212

**Published:** 2018-10-30

**Authors:** Stella Pegoraro Alves, Márcia de Azevedo Frank, Denise Bueno

**Affiliations:** 1 Programa de Pós-Graduação em Assistência Farmacêutica, Faculdade de Farmácia, Universidade Federal do Rio Grande do Sul, Porto Alegre, RS, Brazil.; 2 Serviço de Farmácia, Hospital de Clínicas de Porto Alegre, Porto Alegre, RS, Brazil.

**Keywords:** Drug utilization, Pharmaceutical services, Pharmaceutical preparations, Cystic fibrosis, Child, Caregivers, Uso de medicamentos, Assistência farmacêutica, Preparações farmacêuticas, Fibrose cística, Criança, Cuidadores

## Abstract

**Objective:**

To describe the drug utilization profile used by pediatric cystic fibrosis patients.

**Methods:**

A transversal study comprising the analysis of records and interviews with caregivers of pediatric patient in a reference center of Southern Brazil. We collected information about patients’ clinical condition, medication used and household therapy.

**Results:**

Out of 78 patients participating in the study, prevailing characteristics were: female, self-declared white color, mutation F508del and countryside resident. Forty-three patients had health monitoring exclusively in the hospital’s outpatient division. We analyzed 509 prescribed medication (6.5 medication/patient). The caregiver acknowledged the correct indication in 83% of cases. Patients with pulmonary complications and diseases and/or comorbities related to the cystic fibrosis had an increased quantity of prescribed medication. Vitamins, pancreatic enzymes, hypertonic saline solution, dornase alpha, acid ursodesoxicolic and inhalation antibiotics were most commonly prescribed. Out of the sum of medication, 265 (52.1%) were registered in the *Relação Nacional de Medicamentos Essenciais* , 26.7% were registered in the basic component and 25.4% were registered in the specialized component of pharmaceutical assistance. Seventy-four interviewees informed difficulty in the acquisition of at least one prescribed medication. Most of the reports acknowledge the State Health Department as the place to find and receive medication for cystic fibrosis.

**Conclusion:**

This study allowed reaching a deeper understanding about therapy, caring needed with patients with cystic fibrosis, highlighting to implement strategies that might contribute to enhance life quality and to execute the patients’ therapy plan.

## RESUMO

**Objetivo:**

Descrever o perfil de medicamentos utilizados por pacientes pediátricos com fibrose cística.

**Métodos:**

Estudo transversal com análise de prontuários e entrevista com cuidadores de pacientes pediátricos com fibrose cística atendidos em ambulatório de um centro de referência na Região Sul do Brasil. Foram coletadas informações sobre os dados clínicos dos pacientes, medicamentos utilizados e tratamento domiciliar.

**Resultados:**

Dentre os 78 participantes do estudo, foram predominantes pacientes do sexo feminino, cor autodeclarada branca, com mutação F508del e procedentes do interior do Estado. Possuíam acompanhamento de saúde exclusivamente no ambulatório do hospital 43 pacientes. Foram analisados 509 medicamentos prescritos (6,5 medicamentos/paciente). A indicação correta do medicamento foi reconhecida pelo cuidador em 83,3% dos relatos. Pacientes com complicação pulmonar e doenças e/ou comorbidades associadas à fibrose cística tiveram mais medicamentos prescritos. Vitaminas, enzimas pancreáticas, solução salina hipertônica, alfadornase, ácido ursodesoxicólico e antibióticos inalatórios foram os medicamentos mais prescritos. Destes medicamentos 265 (52,1%) pertenciam à Relação Nacional de Medicamentos Essenciais, sendo 26,7% do componente básico e 25,4% pertencentes ao componente especializado da assistência farmacêutica. Referiram dificuldades para adquirir pelo menos um medicamento prescrito 74 entrevistados. Os relatos, em sua maioria, reconheceram a Secretaria Estadual de Saúde como local de fornecimento dos medicamentos para fibrose cística.

**Conclusão:**

O estudo permitiu maior conhecimento sobre o tratamento e o cuidado necessário em relação ao portador de fibrose cística, observando a demanda da implementação de estratégias que possam contribuir para melhoria da qualidade de vida e o cumprimento do plano terapêutico destes pacientes.

## INTRODUCTION

Cystic fibrosis (CF), or mucoviscidosis, is a lethal autosomal recessive genetic disease that is very common among Caucasians and is characterized by the abnormal production and function of the gene cystic fibrosis transmembrane conductance regulator (CFTR). Cystic fibrosis is multisystemic and affects CFTR-expressing organs and tissues, including the respiratory and gastrointestinal systems, liver and secretory cells.^(^ ^1^ ^,^ ^2^ ^)^

This disease requires intensive control and treatment with ingested and inhaled medication, nebulization, antibiotics, pancreatic enzymes, dietary supplements, and respiratory physical therapy techniques. These different types of care are provided several times per day to yield their beneficial effects.^(^ ^1^ ^,^ ^3^ ^)^ The survival of CF patients depends on several factors, such as unrestricted access to medication and treatment components, and proper instruction of family members and caregivers about the disease and therapy dynamics.^(^ ^4^ ^)^

The data obtained from patients’ treatment routine and dynamic, such as doses and frequency of medications, the means or difficulties to obtain them, and the knowledge of how to use them, allow a multidisciplinary team to define strategies that can positively affect the quality of care, thus avoiding unnecessary readmissions.^(^ ^5^ ^,^ ^6^ ^)^

In CF cases, by acknowledging the importance of prescription and use of these medications we can promote stewardship and improvement of patient care.^(^ ^7^ ^)^

## OBJECTIVE

To describe the profile of medications used by pediatric cystic fibrosis patients.

## METHODS

This is a descriptive cross-sectional study conducted with caregivers of CF patients treated at the pediatric pulmonology outpatient clinic of a reference center for CF, at a university hospital in the Brazilian State of Rio Grande do Sul. This was one of the three reference centers for CF in that state and was open twice a week. The frequency of visits depended on the patient’s clinical status. The patients were seen either monthly or every two months for treatment control and review by a multiprofessional team that included physicians, nurses, dietitians, physical therapists, physical educators, psychologists, social workers and pharmacists. The healthcare team took turns while the patient was in the clinic. This measure was adopted to avoid contamination by multiresistant bacteria, since many of these patients already showed colonization by these pathogens.

We randomly chose respondents since this was a pediatric unit. The selected caregivers were invited by the pharmacist to participate in an interview, which was conducted during one single meeting. The study included caregivers that were accompanying patients with a confirmed CF diagnosis, seen at the outpatient clinic between December 2014 and May 2015. Caregivers who did not agree to sign the informed consent were excluded.

For data collection, we used two instruments developed by the authors of this study, which were previously adjusted and applied.^(^ ^4^ ^)^ The first instrument gathered the patients’ clinical data obtained through an online chart. The second was applied during the pharmaceutical consultation, where caregivers were asked questions in the form of an interview.

The following variables were collected and systematized on a specific database: sex; age; municipality of residence; comprehension of the disease; mutations; presence of respiratory complications, pancreatic insufficiency and other associated diseases; places of treatment outside the outpatient clinic; nutritional diagnosis; feeding route; person in charge of treatment; bacteriology of sputum; and physical therapy and physical exercise.

Caregivers answered questions about data related to medication. All data were acquired through interviews. The caregivers were asked about way of use; dose; frequency; administration route and how they understand the therapeutic indication; means of acquisition; and difficulty complying with the plan of care. Their answers were recorded and generated the data for this study.

To evaluate understanding of CF, caregivers answered the following questions: What do you know about CF? What are the symptoms? What are the consequences of CF? How important is it to correctly follow the treatment plan? Obtained answers were classified as ‘good’, ‘regular’, or ‘bad’.

Medications were classified according to the Anatomical Therapeutic Chemical (ATC) classification system, according to the Brazilian National Relation of Essential Medicines (Rename, abbreviation in Portuguese) 2014/2015,^(^ ^8^ ^)^ and the list of medications from the State Health Department of the State of Rio Grande do Sul (SES/RS - *Secretaria Estadual de Saúde do Rio Grande do Sul* ).^(^ ^9^ ^)^

All data obtained were stored on the software Excel and analyzed with the software Statistical Package for the Social Sciences (SPSS), version 22.0. We used the Kolmogorov-Smirnov test to evaluate the normality of variables, through which we observed they presented an asymmetrical distribution; therefore we decided to express these variables through their medians. Categorical variables were described by absolute and relative frequencies, and the chi-square test was used to associate them and to evaluate their associations. For continuous variables, we used the Mann-Whitney test. To evaluate differences between three or more groups, we used the Kruskal-Wallis test. For all tests, significance level was set at p<0.05.

The study was approved by the Research Ethics Committee of the Research and Graduate Group of the hospital where the study was conducted, under protocol number 802.201, CAAE: 33940114.8.0000.5327.

## RESULTS

In the period of the study, the reference center had, on their records, 119 patients with confirmed CF diagnosis. We analyzed data from 78 patients (65.5%). Most patients were female (53.8%), Caucasian (98.7%), and came from the countryside of the State of Rio Grande do Sul ( Table 1 ). The most common CF mutation was heterozygous for F508del (37.7%). The age group ranged between 4 months to 21 years and 5 months (median of 9.7 years). About 40% of patients had their diagnosis confirmed within their first month of life. One patient died during the study.

**Table 1:** General characteristics of patients

Characteristics	n (%)
Sex	
Female	42 (53.8)
Male	36 (46.2)
Origin	
Countryside of the State of Rio Grande do Sul	52 (66.7)
Metropolitan region	14 (17.9)
Capital city	8 (10.3)
Other states	4 (5.1)
Follow up	
Exclusively outpatients clinic	43 (55.1)
Other healthcare services	35 (44.9)
Caregiver	
Mother	72 (92.3)
Grandmother	2 (2.6)
Father	1 (1.3)
No caregiver/responsible person	3 (3.8)
Responsible for treatment	
Caregiver	55 (70.6)
Caregiver and patient	20 (25.6)
Patient	3 (3.8)

Of all the patients, 43 (53.8%) were followed up exclusively at the hospital outpatient clinic. The others were also seen at other healthcare facilities and organizations.

Associated diseases and/or comorbidities were identified in 33 patients (42.3%), and the most frequenty described was chronic liver disease (16.7%), followed by *diabetes mellitus* , nasal polyps, volume depletion, constipation and allergic rhinitis. Over half the patients (56.4%) presented some form of pulmonary complication, with the most frequent being digital clubbing (47.4%), followed by bronchiectasis (34.6%). Pancreatic insufficiency was present in 89.7% of patient charts and, 35.9% of them mentioned alterations in the liver score (>3). Information about the liver score was not included in the chart of 10.3% of patients (n=8).

In the pharmacotherapeutic analysis of the medications prescribed per patient, there were 2 to 16 drugs prescribed, with a mean of 6.5 medications/patients, totaling up 509 prescribed medicines. However, respondents only understood the purpose or how to use 424 medications (83.3%).

The values in table 2 show an association between the patients’ clinical variables and the prescribed medications. Patients with pulmonary complications and/or diseases associated to CF had more prescribed medications.

**Table 2:** Clinical variables of patients, per prescribed medications

Variables	Prescribed medications (median)	p value
Pulmonary complication		
No	5	<0.001*
Yes	7	
Pancreatic insufficiency		
No	5	0.069*
Yes	6	
Eutrophy		
No	6	0.851*
Yes	6	
Disease and/or comorbidity		
No	6	0.003*
Yes	7	
Exclusive outpatient follow-up		
No	6	0.935*
Yes	6	
Difficulty in treatment		
No	6	0.051*
Yes	7	
Responsible for treatment		
Caregiver	6	0.096^†^
Patient	8	
Caregiver and patient	7	

* p value for Mann-Whitney test; ^†^ p value for Kruskal-Wallis test.

Patients with pancreatic insufficiency (p=0.006), associated diseases (p=0.005) and those who described difficulties in conducting the treatment (p=0.031) showed greater knowledge about the use of medications than those without these characteristics.

Among the prescribed medications, 265 are included in Rename: 26.7% of them are in their list of “Basic Components of Pharmaceutical Care”, and 25.4% are in their list of “Specialized Components of Pharmaceutical Care”.

Of the medications prescribed to the patients in our study, 289 are used by SES/RS: 12% are on their list of “Basic Components of Pharmaceutical Care”, 24.7% are on their list of “Specialized Components of Pharmaceutical Care”, 34.8% are on their list of “Special Medications”, and 28.5% were not included on the list of medications selected.

The medications to treat the digestive tract and metabolism were the most often prescribed (45.8%), according to the ATC, followed by medications for the respiratory system (35.4%) and anti-infective drugs (14.5%). Vitamins were the most frequently prescribed drugs, in the isolated and multivitamin forms ( Table 3 ).

**Table 3:** Most frequently prescribed drugs

Drug	Number	Percentage in relation to total number of prescribed drugs	Percentage in relation to total number of patients
Multivitamin 1*	75	14.7	96.1
Pancreatic enzymes	72	14.1	92.3
Hypertonic saline solution	66	12.9	84.6
Dornase alfa	53	10.4	68.0
Ursodesoxicolic acid	33	6.5	42.3
Polymyxin E	28	5.5	35.9
Tobramycin	23	4.5	29.5
Phenoterol	20	3.9	25.6
Azithromycin	16	3.1	20.5
Multivitamin 2†	16	3.1	20.5
Budesonide	14	2.7	17.9
Salbutamol	10	2.0	12.8
Omeprazole	9	1.8	11.5
Montelucaste	8	1.6	10.2
Insulins	7	1.5	9.0
Vitamin E	7	1.5	9.0
Others	52	10.2	66.7

* Ingredients: vitamins A, B6, C, D, E and K1; ^†^ ingredients: vitamins A, B1, B2, B3, B5, B6, B8, C, D2 and E.

Oral (n=233; 52.7%) and inhaled (n=203; 39.9%) medicines were the most frequently prescribed, followed by intranasal medications (4.1%), via gastrostomy (2.1%) and subcutaneous medications (1.4%).

**Figure 1:**
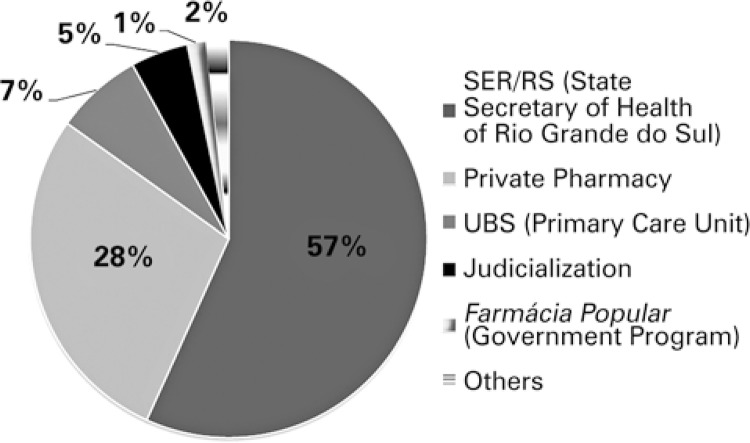
Locations where medications were acquired

Figure 1 shows the locations where the medications for the patients were acquired. Another 10 respondents said they obtained the drugs using different ways: 6 said they purchase the medication at the hospital in their hometown, 2 received the medication from the CF outpatient clinic, 1 patient received it from a private pediatrician’s office, and 1 received it from the hospital where his caregiver worked.

Of the respondents, 64 (82%) reported difficulties in obtaining at least one of the medications, and that varied between 1 and 5 medications of a total of 101 medicines (mean: 1.5 medications/patient). The main cause of patients not having access to a certain medication was shortage at healthcare facilities. According to the respondents’ reports, the medications most difficult to obtain were multivitamin 1, polymyxin E, pancreatic enzymes, dornase alfa, azithromycin, ursodeoxycholic acid, and tobramycin.

The non-pharmacological treatment was mentioned as important in the reports. All respondents said the patients did physical therapy at home and 85.9% did it at least twice a day. Moreover, 62% of patients did some type of physical exercise, 31.2% of whom did it at school only (during physical education classes).

Regarding nutritional status, most patients (52.6%) were classified as eutrophic. They used calorie supplements, liquid diets and diets with complex carbohydrates, such as maltodextrin, in 59% of cases. One patient was fed only through gastrostomy. Two patients, who used gastrotomy and tube feeding, had a complementary feeding pathway to the oral pathway.

Methicillin-sensitive *Staphylococcus aureus* (MSSA) was the most prevalent (64.1%) agent in patient’s sputum, followed by *Pseudomonas aeruginosa* (34.6%) and *Burkholderia cepacia* (20.5%). Of the exams showing *Pseudomonas aeruginosa,* 25.9% of cases presented its mucoid form.

For 92.3% of accompanied patients, the mother was the main caregiver. Full understanding of CF, its symptoms, consequences and the importance of treatment was observed in 65.4% of cases.

The results showed the relation between knowledge about the disease and clinical data of patients. Those who were followed up in other health organizations (n=27; 77.1%; p=0.008) and who presented pancreatic insufficiency (n=49; 70%; p=0.027) showed a better understanding of the disease in comparison to patients with a good pancreatic state who are treated exclusively at the outpatient clinic.

## DISCUSSION

Most of the study subjects were female, Caucasian, from the countryside of the State of Rio Grande do Sul, with a F508del mutation and the presence of MSSA in the sputum. These data were similar to those observed in studies conducted in this^(^ ^10^ ^,^ ^11^ ^)^ and other states of Brazil.^(^ ^12^ ^,^ ^13^ ^)^

Respondents, in general, showed good knowledge of the disease. A study conducted at a reference center in the United Kingdom also found that adult CF patients showed good general knowledge about the disease.^(^ ^14^ ^)^

Some patients were followed up exclusively in the outpatient clinic where the study was conducted, and have a visible bond with the clinic medical staff. The accounts given by caregivers showed the difficulty of being followed up in another health setting, besides the outpatient clinic. Professionals and services, particularly in the countryside, are faced with limitations and, at times, a lack of training on how to address patients with chronic diseases.

Oftentimes, CF patients are referred to other services – regardless of level of care - and the referral does not generally include detailed orientation on how to carry on with the plan of care. Since the supply of medical appointments is not always appropriate, the patient is referred to a specialist under the assumption that only this physician will be able to provide the best treatment. As a consequence, the bond is established only with the outpatient of the CF reference center.^(^ ^15^ ^)^

A few conditions are necessary for a good transition within the healthcare complexity levels. Some difficulties may result in worsening of the patient’s condition and in overload for more complex units of the Brazilian Public Health System (SUS – *Sistema Único de Saúde* ).^(^ ^16^ ^)^ Therefore, it is paramount that healthcare professionals are trained on how to address patients at low or medium complexity services, to avoid overloading high complexity services. In this study, patients who were treated not only at the reference center have a better understanding of CF, which certainly improves compliance to treatment and quality of life.

We also observed the importance of the interval between CF diagnosis confirmation and starting of treatment. The analysed patients presented an association between pulmonary complications and diseases and/or comorbidities. Since this is a pediatric center, it is worth mentioning the impact of knowledge on the disease and prognosis of patients.

Mothers were the main caregivers, and, in a few cases, patient care was shared with another family member. Similar situations are shown in the literature.^(^ ^17^ ^,^ ^18^ ^)^ The company of a caregiver has a positive impact in monitoring of the plan of care.

The respondents were able to recognize the purpose of most medications prescribed. The correct use of the medicines was more often reported by caregivers of patients with complications and/or comorbidities. Another study, conducted in the internal medicine outpatient clinic of the same hospital, to evaluate the level of information given to patients about the medications prescribed, demonstrated the information was accurate in most cases.^(^ ^19^ ^)^ Oenning et al., evaluated knowledge of patients during medical appointments and dispensing, and found values of over 90%.^(^ ^20^ ^)^

Poor digestion and/or poor absorption of fat and vitamins affect the patient’s energy and nutritional balance, which is directly related to a decline of pulmonary function and of quality of life.^(^ ^21^ ^)^ This is why most prescribed medications, according to ATC, belong to the pharmacological group of digestive tract and metabolism drugs, such as vitamins and pancreatic enzymes. The next most prescribed drugs are for the respiratory tract, which is another system that is affected in most CF patients. That explains why multivitamins are widely prescribed.

Pancreatic enzymes were prescribed to almost all patients, which is in accordance with data from the Brazilian Cystic Fibrosis Registry (REBRAFC)^(^ ^22^ ^)^ and from the Cystic Fibrosis Foundation (CFF):^(^ ^23^ ^)^ 81.2% and 87.3%, respectively. European data from 2010 presented the prescription of pancreatic enzymes for over 90% of cases in Serbia, Denmark and Russia, and for approximately 85% of patients in Sweden, Lithuania, Switzerland and Greece.^(^ ^24^ ^)^ Rizzo et al., conducted studies in two reference center for CF and showed pancreatic enzymes prescribed to 96% and 83.6% of patients. Scattolin et al., found that 90.3% of patients were undergoing enzyme replacement therapy.^(^ ^10^ ^,^ ^11^ ^)^

Dornase alfa was less often prescribed drug, likewise in other Brazil investigations,^(^ ^10^ ^,^ ^11^ ^)^ as compared to data from the CFF.^(^ ^23^ ^)^ There are studies associating the use of dornase alfa to a reduced risk of death, improved pulmonary function, and decreased pulmonary exacerbations.^(^ ^3^ ^)^ Wark et al., highlighted that treatment with this mucolytic agent is relatively expensive, and its use is limited in most countries. This fact would explain the abovementioned discrepancy, and so the therapeutic alternative is to use hypertonic saline solution.^(^ ^25^ ^)^

The frequency of hypertonic saline solution prescriptions (84.6%) was higher in comparison data to data from CFF (65.7%) and from the UK Cystic Fibrosis Registry (26.1%).^(^ ^23^ ^,^ ^26^ ^)^ In addition to its low cost, the preparation of hypertonic saline solution is simpler and can be done by the caregiver and/or the patient, as many of our respondents said they do. As an alternative, the hypertonic saline solution can be purchased in its prepared form at private drugstores. Its use helps reducing pulmonary exacerbations and the use of antibiotics, and improves the patient’s quality of life.^(^ ^25^ ^)^

Ursodeoxycholic acid, which protects the liver, was prescribed to almost half the patients, since chronic liver disease is the most common comorbidity among these patients, and 35% of them presented alterations in the liver score. Similar prescription frequencies were found in at least six European countries.^(^ ^24^ ^)^ Another study that had previously characterized the population of the same outpatient clinic found that 55.5% of patients had been prescribed ursodeoxycholic acid.^(^ ^11^ ^)^

The use of inhaled tobramycin to eradicate *Pseudomonas aeruginosa,* is higher in the data from the CFF (69.8%). This difference can be explained by the higher frequency of *Pseudomonas aeruginosa* colonization among North-American patients.^(^ ^23^ ^)^ Polymyxin E, an alternative treatment, was prescribed to 35.9% patients, which is similar to the number found in registries from Brazil and the United Kingdom.^(^ ^22^ ^,^ ^26^ ^)^ In the United States, polymyxin has not been approved as an inhaled antibiotic.

As to the acquisition of medications, a little more than 50% of them were included in the Rename,^(^ ^8^ ^)^ which is a reference for SUS that should guide the supply, prescription and dispensing of medications.

States and municipalities have political-administrative autonomy and can define their list of essential medications based on different epidemiological profiles to meet the demands of each location. This can often generate confusion among caregivers, who do not know which medication they can find at health organizations.^(^ ^27^ ^)^ Such situation was observed in this study. The respondents mentioned up to nine different health services and organizations they had visited, including situations in which they had to go to three to four sites to obtain all medications required.

Brazil has no specific policy for rare genetic diseases and care delivered to these patients requires much improvement.^(^ ^28^ ^)^ Cystic fibrosis is addressed in two Clinical Protocols and Therapeutic Guidelines: pulmonary manifestations with dornase alfa, and pancreatic insufficiency with enzyme replacement therapy. Both medications are in the specialized component list of the Brazilian Ministry of Health and are co-financed and distributed by the federal and state governments. According to the SES/RS classification, most medications were in the special components list.^(^ ^9^ ^)^

The State of Rio Grande do Sul, in addition to its list of specialized components, distributes medications from a complementary list that includes other medications, diets and dietary supplements. This list was created in 2010 to maintain and expand CF patients’ access to medications.^(^ ^29^ ^)^ Most drugs are distributed by the SUS which may hinder access in situations of shortage and difficulty in obtaining the drugs in the private sector.^(^ ^30^ ^)^ This problem was mentioned by most respondents, who said they had difficulties obtaining medications, especially multivitamins (a medicine that is imported by SES/RS).

Our findings can help create public policies that benefit pediatric CF patients by promoting assurance of orientation and access to the medications needed for their treatment, regardless of the health organization they visit. It is paramount to promote strategies that can improve public policies, with educational approaches that view the patient as the center of the care process and allow them to voice their questions, difficulties, opinions and experiences related to the treatment. That would minimize problems related to compliance to treatment, decrease unnecessary hospital admissions and ensure a better quality of life for these children.

## CONCLUSION

The data gathered have given us more information about the treatment, care and needs of cystic fibrosis patients. Considering many complications associated with cystic fibrosis, patients are generally on several medications. Most of them take pancreatic enzymes, vitamins, mucolytics, and antimicrobial agents after being diagnosed. Cystic fibrosis patients often have difficulties in complying with treatment, especially drug therapy, for lack of information and problems obtaining the medications, even though half of them are in Rename. cystic fibrosis patients require intense care from their families, healthcare team and organizations that follow them up.
